# Goodbye Hartmann trial: a prospective, international, multicenter, observational study on the current use of a surgical procedure developed a century ago

**DOI:** 10.1186/s13017-024-00543-w

**Published:** 2024-04-16

**Authors:** Gennaro Perrone, Mario Giuffrida, Fikri Abu-Zidan, Vitor F. Kruger, Marco Livrini, Gabriele Luciano Petracca, Giorgio Rossi, Antonio Tarasconi, Brian W. C. A. Tian, Elena Bonati, Ricardo Mentz, Federico N. Mazzini, Juan P. Campana, Elisabeth Gasser, Reinhold Kafka-Ritsch, Daniel M. Felsenreich, Christopher Dawoud, Stefan Riss, Carlos Augusto Gomes, Felipe Couto Gomes, Ricardo Alessandro Teixeira Gonzaga, Cassio Alfred Brattig Canton, Bruno Monteiro Pereira, Gustavo P. Fraga, Leticia Gonçalves Zem, Vinicius Cordeiro-Fonseca, Renato de Mesquita Tauil, Boyko Atanasov, Nikolay Belev, Nikola Kovachev, L. Juan José Meléndez, Ana Dimova, Stefan Dimov, Zdravko Zelić, Goran Augustin, Branko Bogdanić, Trpimir Morić, Elie Chouillard, Melinda Bajul, Belinda De Simone, Yves Panis, Francesco Esposito, Margherita Notarnicola, Lelde Lauka, Anna Fabbri, Hassen Hentati, Iskander Fnaiech, Venara Aurélien, Marie Bougard, Maxime Roulet, Zaza Demetrashvili, Irakli Pipia, Giorgi Merabishvili, Konstantinos Bouliaris, Georgios Koukoulis, Christos Doudakmanis, Sofia Xenaki, Emmanuel Chrysos, Stamatios Kokkinakis, Panteleimon Vassiliu, Nikolaos Michalopoulos, Ioannis Margaris, Aristotelis Kechagias, Konstantinos Avgerinos, Jevgeni Katunin, Eftychios Lostoridis, Eleni-Aikaterini Nagorni, Antonio Pujante, Francesk Mulita, Ioannis Maroulis, Michail Vailas, Athanasios Marinis, Ioannis Siannis, Eirini Bourbouteli, Dimitrios K. Manatakis, Nikolaos Tasis, Vasileios Acheimastos, Sotiropoulou Maria, Kapiris Stylianos, Harilaos Kuzeridis, Dimitrios Korkolis, Evangelos Fradelos, George Kavalieratos, Thalia Petropoulou, Andreas Polydorou, Ioannis Papacostantinou, Tania Triantafyllou, Despina Kimpizi, Dimitrios Theodorou, Konstantinos Toutouzas, Alexandros Chamzin, Maximos Frountzas, Dimitrios Schizas, Ioannis Karavokyros, Athanasios Syllaios, Alexandros Charalabopoulos, Maria Boura, Efstratia Baili, Orestis Ioannidis, Lydia Loutzidou, Elissavet Anestiadou, Ioannis Tsouknidas, Georgios Petrakis, Eleni Polenta, Lovenish Bains, Rahul Gupta, Sudhir K. Singh, Archana Khanduri, Miklosh Bala, Asaf Kedar, Marcello Pisano, Mauro Podda, Adolfo Pisanu, Gennaro Martines, Giuseppe Trigiante, Giuliano Lantone, Antonino Agrusa, Giuseppe Di Buono, Salvatore Buscemi, Massimiliano Veroux, Rossella Gioco, Gastone Veroux, Luigi Oragano, Sandro Zonta, Federico Lovisetto, Carlo V. Feo, Antonio Pesce, Nicolò Fabbri, Giulio Lantone, Fabio Marino, Fabrizio Perrone, Leonardo Vincenti, Vincenzo Papagni, Arcangelo Picciariello, Stefano Rossi, Biagio Picardi, Simone Rossi Del Monte, Diego Visconti, Giulia Osella, Luca Petruzzelli, Giusto Pignata, Jacopo Andreuccetti, Rossella D’Alessio, Massimo Buonfantino, Eleonora Guaitoli, Stefano Spinelli, Gianluca Matteo Sampietro, Carlo Corbellini, Leonardo Lorusso, Alice Frontali, Isabella Pezzoli, Alessandro Bonomi, Andrea Chierici, Christian Cotsoglou, Giuseppe Manca, Antonella Delvecchio, Nicola Musa, Massimiliano Casati, Laface Letizia, Emmanuele Abate, Giorgio Ercolani, Fabrizio D’Acapito, Leonardo Solaini, Gianluca Guercioni, Simone Cicconi, Diego Sasia, Felice Borghi, Giorgio Giraudo, Giuseppe Sena, Pasquale Castaldo, Eugenia Cardamone, Giuseppe Portale, Matteo Zuin, Ylenia Spolverato, Marialusia Esposito, Roberta Maria Isernia, Maria Di Salvo, Romina Manunza, Giuseppe Esposito, Marcello Agus, Emanuele Luigi Giuseppe Asti, Daniele Tiziano Bernardi, Tommaso Panici Tonucci, Davide Luppi, Massimiliano Casadei, Stefano Bonilauri, Angela Pezzolla, Annunziata Panebianco, Rita Laforgia, Maurizio De Luca, Monica Zese, Dario Parini, Elio Jovine, Giuseppina De Sario, Raffaele Lombardi, Giovanni Aprea, Giuseppe Palomba, Marianna Capuano, Giulio Argenio, Gianluca Orio, Mariano Fortunato Armellino, Marina Troian, Martina Guerra, Carlo Nagliati, Alan Biloslavo, Paola Germani, Giada Aizza, Igor Monsellato, Ali Chaouki Chahrour, Gabriele Anania, Cristina Bombardini, Francesco Bagolini, Gabriele Sganga, Pietro Fransvea, Valentina Bianchi, Paolo Boati, Francesco Ferrara, Francesco Palmieri, Pasquale Cianci, Domenico Gattulli, Enrico Restini, Nicola Cillara, Alessandro Cannavera, Gabriela Elisa Nita, Jlenia Sarnari, Francesco Roscio, Federico Clerici, Ildo Scandroglio, Stefano Berti, Alessandro Cadeo, Alice Filippelli, Luigi Conti, Carmine Grassi, Gaetano Maria Cattaneo, Marina Pighin, Davide Papis, Giovanni Gambino, Vanessa Bertino, Domenico Schifano, Daniela Prando, Luisella Fogato, Fabio Cavallo, Luca Ansaloni, Roberto Picheo, Nicholas Pontarolo, Norma Depalma, Marcello Spampinato, Stefano D’Ugo, Luca Lepre, Michela Giulii Capponi, Rossella Domenica Campa, Giuliano Sarro, Vincenza Paola Dinuzzi, Stefano Olmi, Matteo Uccelli, Davide Ferrari, Marco Inama, Gianluigi Moretto, Michele Fontana, Francesco Favi, Erika Picariello, Alessia Rampini, Andrea Barberis, Antonio Azzinnaro, Alba Oliva, Luigi Totaro, Ilaria Benzoni, Valerio Ranieri, Gabriella Teresa Capolupo, Filippo Carannante, Marco Caricato, Maurizio Ronconi, Silvia Casiraghi, Giovanni Casole, Desire Pantalone, Giovanni Alemanno, Maximilian Scheiterle, Marco Ceresoli, Marco Cereda, Chiara Fumagalli, Federico Zanzi, Stefano Bolzon, Enrico Guerra, Francesca Lecchi, Paola Cellerino, Antonella Ardito, Rosa Scaramuzzo, Andrea Balla, Pasquale Lepiane, Nicola Tartaglia, Antonio Ambrosi, Giovanna Pavone, Gian Marco Palini, Simone Veneroni, Gianluca Garulli, Claudio Ricci, Beatrice Torre, Iris Shari Russo, Matteo Rottoli, Marta Tanzanu, Angela Belvedere, Marco Milone, Michele Manigrasso, Giovanni Domenico De Palma, Micaela Piccoli, Gianmaria Casoni Pattacini, Stefano Magnone, Paolo Bertoli, Michele Pisano, Paolo Massucco, Marco Palisi, Andrea-Pierre Luzzi, Francesco Fleres, Guglielmo Clarizia, Alessandro Spolini, Yoshiro Kobe, Takayuki Toma, Fumihiko Shimamura, Robert Parker, Sinkeet Ranketi, Mercy Mitei, Saulius Svagzdys, Henrikas Pauzas, Justas Zilinskas, Tomas Poskus, Marius Kryzauskas, Matas Jakubauskas, Andee Dzulkarnaen Zakaria, Zaidi Zakaria, Michael Pak-Kai Wong, Asri Che Jusoh, Muhammad Nazreen Zakaria, Daniel Rios Cruz, Aurea Barbara Rodriguez Elizalde, Alejandro Bañon Reynaud, Edgard Efren Lozada Hernandez, Jose maria Victor Palomo Monroy, Diego Hinojosa-Ugarte, Martha Quiodettis, María Esther Du Bois, José Latorraca, Piotr Major, Michał Pędziwiatr, Magdalena Pisarska-Adamczyk, Maciej Walędziak, Andrzej Kwiatkowski, Łukasz Czyżykowski, Silvia Dantas da Costa, Bela Pereira, Ana Rita Oliveira Ferreira, Filipe Almeida, Ricardo Rocha, Carla Carneiro, Diego Pita Perez, João Carvas, Catarina Rocha, Cátia Ferreira, Rita Marques, Urânia Fernandes, Pedro Leao, André Goulart, Rita Gonçalves Pereira, Sara Daniela Direito Patrocínio, Nuno Gonçalo Gonçalves de Mendonça, Maria Isabel Cerqueira Manso, Henrique Manuel Cardoso Morais, Paulo Sebastião Cardoso, Valentin Calu, Adrian Miron, Elena Adelina Toma, Mahir Gachabayov, Abakar Abdullaev, Andrey Litvin, Taras Nechay, Alexander Tyagunov, Anvar Yuldashev, Alison Bradley, Michael Wilson, Arpád Panyko, Zuzana Látečková, Vladimír Lacko, Dusan Lesko, Marek Soltes, Jozef Radonak, Victor Turrado-Rodriguez, Roser Termes-Serra, Xavier Morales-Sevillano, Pierfrancesco Lapolla, Andrea Mingoli, Gioia Brachini, Maurizio Degiuli, Silvia Sofia, Rossella Reddavid, Andrea de Manzoni Garberini, Angelica Buffone, Eduardo Perea del Pozo, Daniel Aparicio-Sánchez, Sandra Dos Barbeito, Mercedes Estaire-Gómez, Rebeca Vitón-Herrero, Mª de los Ángeles Gil Olarte-Marquez, José Gil-Martínez, Felipe Alconchel, Tatiana Nicolás-López, Aida Cristina Rahy-Martin, María Pelloni, Raquel Bañolas-Suarez, Fernando Mendoza-Moreno, Francisca García-Moreno Nisa, Manuel Díez-Alonso, María Elisa Valle Rodas, María Carmona Agundez, María Inmaculada Pérez Andrés, Claudia Cristina Lopes Moreira, Aintzane Lizarazu Perez, Iñigo Augusto Ponce, Ana María González-Castillo, Estela Membrilla-Fernández, Silvia Salvans, Mario Serradilla-Martín, Pablo Sancho Pardo, Daniel Rivera-Alonso, Jana Dziakova, Jose Mugüerza Huguet, Naila Pagès Valle, Enrique Colás Ruiz, Cristina Rey Valcárcel, Cristina Ruiz Moreno, Yeniffer Tatiana Moreno Salazar, Juan Jesús Rubio García, Silvia Sevila Micó, Joaquín Ruiz López, Silvia Pérez Farré, Maite Santamaria Gomez, Nuria Mestres Petit, Alberto Titos-García, Jose Manuel Aranda-Narváez, Laura Romacho-López, Luis Sánchez-Guillén, Veronica Aranaz-Ostariz, Marina Bosch-Ramírez, Aleix Martínez-Pérez, Elías Martínez-López, Juan Carlos Sebastián-Tomás, Granada Jimenez-Riera, Javier Jimenez-Vega, Jose Aurelio Navas Cuellar, Andrea Campos-Serra, Anna Muñoz-Campaña, Raquel Gràcia-Roman, Javier Martínez Alegre, Francisca Lima Pinto, Sara Nuñez O’Sullivan, Francisco Blanco Antona, Beatriz Muñoz Jiménez, Jaime López-Sánchez, Zahira Gómez Carmona, Rocio Torres Fernández, Isabel Blesa Sierra, Laura Román García de León, Verónica Polaino Moreno, Eva Iglesias, Paola Lora Cumplido, Altea Arango Bravo, Ignacio Rey Simó, Carlota López Domínguez, Aloia Guerreiro Caamaño, Rafael Calleja Lozano, Manuel Durán Martínez, Álvaro Naranjo Torres, Javier Tomas Morales Bernaldo de Quiros, Gianluca Pellino, Miriam Moratal Cloquell, Elsa García Moller, Sami Jalal-Eldin, Ahmed K. Abdoun, Hytham K. S. Hamid, Varut Lohsiriwat, Aitsariya Mongkhonsupphawan, Oussama Baraket, Karim Ayed, Imed Abbassi, Ali Ben Ali, Houssem Ammar, Ali Kchaou, Ahmed Tlili, Imen Zribi, Elif Colak, Suleyman Polat, Zehra Alan Koylu, Ali Guner, Mehmet Arif Usta, Murat Emre Reis, Baris Mantoglu, Emre Gonullu, Emrah Akin, Fatih Altintoprak, Zulfu Bayhan, Necattin Firat, Arda Isik, Ufuk Memis, Mehmet Bayrak, Yasemin Altıntaş, Yasin Kara, Mehmet Abdussamet Bozkurt, Ali Kocataş, Koray Das, Ahmet Seker, Nazmi Ozer, Semra Demirli Atici, Korhan Tuncer, Tayfun Kaya, Zeynep Ozkan, Onur Ilhan, Ibrahim Agackiran, Mustafa Yener Uzunoglu, Eren Demirbas, Yuksel Altinel, Serhat Meric, Nadir Adnan Hacım, Derya Salim Uymaz, Nail Omarov, Emre Balık, Giovanni D. Tebala, Hany Khalil, Mridul Rana, Mansoor Khan, Charlotte Florence, Christie Swaminathan, Cosimo Alex Leo, Lampros Liasis, Josef Watfah, Ivan Trostchansky, Edward Delgado, Marcelo Pontillo, Rifat Latifi, Raul Coimbra, Sara Edwards, Ana Lopez, George Velmahos, Ander Dorken, Anthony Gebran, Amanda Palmer, Jeffrey Oury, James M. Bardes, Sirivan Suon Seng, Lauren S. Coffua, Asanthi Ratnasekera, Tanya Egodage, Karla Echeverria-Rosario, Isabella Armento, Lena M. Napolitano, Naveen F. Sangji, Mark Hemmila, Jacob A. Quick, Tyler R. Austin, Theodore S. Hyman, William Curtiss, Amanda McClure, Nicholas Cairl, Walter L. Biffl, Hung P. Truong, Kathryn Schaffer, Summer Reames, Filippo Banchini, Patrizio Capelli, Federico Coccolini, Massimo Sartelli, Francesca Bravi, Carlo Vallicelli, Vanni Agnoletti, Gian Luca Baiocchi, Fausto Catena

**Affiliations:** 1 Department of Emergency Surgery, Maggiore Hospital, Parma, Italy; 2 General Surgery Unit, Maggiore Hospital, Parma, Italy; 3 Department of General Surgery, Ospedale Guglielmo da Saliceto, 29100 Piacenza, Italy; 4 Department of Surgery, College of Medicine and Health Sciences, UAE University, Al-Ain, United Arab Emirates; 5 Division of Trauma Surgery, School of Medical Sciences, University of Campinas, Campinas, Brazil; 6 General Surgery Department, UO Chirurgia Generale, ASST Cremona, Cremona, Italy; 7 Department of General Surgery, Singapore General Hospital, Singapore, Singapore; 8 General Surgery Department, Hospital Italiano de Buenos Aires, Buenos Aires, Argentina; 9 Department of Visceral, Transplant and Thoracic Surgery, Medical University of Innsbruck, Innsbruck, Austria; 10 Division of Visceral Surgery, Department of General Surgery, Medical University of Vienna, Vienna, Austria; 11 Faculdade de Medicina, SUPREMA, Hospital Universitario Terezinha de Jesus de Juiz de Fora, Juiz de Fora, MG Brazil; 12 Medical Course, Department of Surgery - Emergency Surgery and Trauma Sector, Padre Albino University Center, Catanduva, Brazil; 13 Grupo Surgical, Campinas, Brazil; 14 Hospital Vivalle, São José Dos Campos, Brazil; 15 UMHAT Eurohospital-Plovdiv/Medical University Plovdiv, Plovdiv, Bulgaria; 16 Trauma and Acute Care Surgeon Hospital Rafael Angel Calderón Guardia, San José, Costa Rica; 17 General Hospital Zabok and Croatian War Veteran Hospital Bracak, Zabok, Croatia; 18 Department of Surgery, University Hospital Centre Zagreb, Zagreb, Croatia; 19 Department of General and Bariatric Surgery, American Hospital in Paris, Paris, France; 20 Emergency and General Minimally Invasive Surgery, Poissy and St Germain Hospital, Poissy, France; 21 Department of Emergency and General Minimally Invasive Surgery, Academic Hospital of Villeneuve St Georges, Villeneuve-Saint-Georges, France; 22 Colorectal Center, Groupe Hospitalier Privé Ambroise Paré-Hartmann, Neuilly/Seine, France; 23 Department of Colorectal Surgery, Pôle Des Maladies de L’Appareil Digestif (PMAD), Beaujon Hospital, Assistance Publique-Hôpitaux de Paris (AP-HP), Paris, France; 24 Hôpital Henri Mondor, Créteil, France; 25 Hôpital Léon Binet, Provins, France; 26 Department of Visceral Surgery CHU Angers, 4 Rue Larrey, 49933 Angers Cedex 9, France; 27 N.Kipshidze Central University Hospital, Tbilisi, Georgia; 28 Surgical Department, General Hospital of Larissa, Larissa, Greece; 29 Department of General Surgery - University Hospital of Heraklion Crete, Crete, Greece; 30 4th Surgical Department “Attikon” University Hospital, Chaidari, Greece; 31 Department of Digestive Surgery, Athens Bioclinic Hospital, Athens, Greece; 32 Kavala General Hospital, Kavala, Greece; 33 Department of Surgery, General University Hospital of Patras, Patras, Greece; 34 Third Department of Surgery, Tzaneio General Hospital, Piraeus, GR Greece; 35 2nd Department of Surgery, Athens Naval and Veterans Hospital, Athens, Greece; 36 Evangelismos General Hospital, Athens, Greece; 37 Surgical Oncology Department of Agios Savvas Anticancer Hospital of Athens, Athens, Greece; 38 Aretaieio University Hospital, Athens, Greece; 39 Department of Surgery, Hippocration General Hospital of Athens, University of Athens, Athens, Greece; 40 1st Propaedeutic Department of Surgery, Hippocratio Hospital of Athens, Athens, Greece; 41 First Department of Surgery, National and Kapodistrian University of Athens, Laikon General Hospital, Athens, Greece; 42 National and Kapodistrian University of Athens, Athens, Greece; 43 4th Department of Surgery, School of Medicine, Faculty of Health Sciences, Aristotle University of Thessaloniki, General Hospital “George Papanikolaou”, Thessaloniki, Greece; 44 2n, Department of Surgery, General Hospital of Chania “St George”, Chania, Greece; 45 Department of Surgery, Maulana Azad Medical College, New Delhi, India; 46 Department of Gastrointestinal Surgery, Synergy Institute of Medical Sciences, Dehradun, India; 47 General Surgery and Trauma Unit Hadassah Hebrew University Medical Center, Jerusalem, Israel; 48 Chirurgia d’Urgenza Policlinico Universitario di MonserratoAzienda Ospedaliero Universitaria di Cagliari, Cagliari, Italy; 49 Chirurgia “M.Rubino” Azienda Ospedaliero Universitaria Policlinico Bari, Bari, Italy; 50 Unit of General and Emergency Surgery, Department of Surgical, Oncological and Oral Sciences (Di.Chir.On.S.), University Hospital Policlinico “P. Giaccone, University of Palermo - Palermo, Palermo, Italy; 51 General Surgery Unit and Organ Transplant Unit, University Hospital of Catania, Catania, Italy; 52 SOC Chirurgia Generale - ASL VCO (Piemonte), Verbania, Italy; 53 Unità Operativa Chirurgia Generale ProvincialeAzienda USL di Ferrara, Ferrara, Italy; 54 Surgery Unit, National Institute of Gastroenterology ‘S. de Bellis’, Research Hospital, Castellana Grotte, BA Italy; 55 Azienda Ospedaliero Universitaria Consorziale Policlinico di Bari - Dept of Emergency and Organ Transplantation, Bari, Italy; 56 Department of General and Emergency Surgery, San Filippo Neri Hospital, Rome, Italy; 57 Chirurgia Generale d’Urgenza e PS - AOU Città della Salute e della Scienza di Torino, Presidio San Giovanni Battista - Molinette, Turin, Italy; 58 Chirurgia Generale 2 ASST Spedali Civili di Brescia, Brescia, Italy; 59 Chirurgia Generale PO Valle d’Itria ASL TA, Martina Franca, Italy; 60 Unità Operativa di Chirurgia Generale Ospedale di Rho - ASST Rhodense, Milan, Italy; 61 General Surgery Unit, Department of Biomedical and Clinical Sciences “L. Sacco”, University of Milan, AAST Fatebenefratelli Sacco, Milan, Italy; 62 General Surgery Unit, University of Milan, ASST Vimercate, Via Santi Cosma e Damiano 16, 20871 Vimercate, Italy; 63 Unità Operativa Complessa di Chirurgia Generale, Presidio Ospedaliero “A. Perrino”, Brindisi, Italy; 64 Ospedale Vittorio Emanuele III Carate Brianza, Carate Brianza, Italy; 65 UOC Chirurgia Generale e Terapie Oncologiche Avanzate Ospedale Morgagni-Pierantoni AUSLRomagna, Via Carlo Forlanini 34, 47121 Forlì, Italy; 66 UOC ChirurgiaOspedale Provinciale “C. E G. Mazzoni” Ascoli Piceno, Area Vasta 5, Regione Marche, Italy; 67 Santa Croce and Carle Hospital, Cuneo, Italy; 68 Dipartimento di Specialità Chirurgiche, Azienda Ospedaliera “Pugliese-Ciaccio” di Catanzaro, Catanzaro, Italy; 69 Department of General Surgery, ULSS 6 Euganea, CittadellaPadua, Italy; 70 Chirurgia Generale Ospedale “Di Venere”, Bari, Italy; 71 Chirurgia d’Urgenza ospedale Brotzu – ARNAS, Palermo, Italy; 72 University of Milan, IRCCS Policlinico San Donato, Milan, Italy; 73 Dipartimento di Chirurgia Generale 2 e D’Urgenza dell’Arcispedale Santa Maria Nuova – Ausl RE IRCCS, Reggio Emilia, Italy; 74 U.O. di Chirurgia Videolaparoscopica della AOU Policlinico di Bari, Bari, Italy; 75 Department of General Surgery, Santa Maria della Misericordia Hospital, AULSS5 Polesana - Rovigo, Rovigo, Italy; 76 Chirurgia A e d’Urgenza IRCCS Ospedale Maggiore Bologna Largo, Bartolo Nigrisoli 2, 40133 Bologna, Italy; 77 UOC Chirurgia Endoscopica - AOU Federico II Di Napoli, Naples, Italy; 78 UOC Chirurgia d’Urgenza AOU San Giovanni di Dio e Ruggi d’Aragona, Salerno, Italy; 79 SC Chirurgia Generale, ASUGI - Ospedale San Giovanni di Dio, Gorizia, Italy; 80 Clinica Chirurgica, Cattinara University Hospital, ASUGI Trieste, Trieste, Italy; 81 SS Antonio e Biagio e Cesare Arrigo Hospital, Alessandria, Italy; 82 University Hospital of Ferrara, Ferrara, Italy; 83 Fondazione Policlinico Universitario A. Gemelli, IRCCS, Roma - Università Cattolica del Sacro Cuore, Rome, Italy; 84 Department of Surgery, San Carlo Borromeo Hospital, ASST Santi Paolo e Carlo, Milan, Italy; 85 Department of Surgery and Traumatology-General Surgery Unit, “Lorenzo Bonomo Hospital”-ASL BAT, Andria, Italy; 86 Chirurgia Generale PO Santissima Trinità - ASL Cagliari, Cagliari, Italy; 87 Chirurgia GeneraleOspedale Sant’Anna di AUSL di Reggio Emilia, Reggio Emilia, Italy; 88 Division of General Surgery, ASST Valle Olona, Busto Arsizio, Italy; 89 S.C. Chirurgia Generale, S. Andrea Hospital - ASL 5, La Spezia, Italy; 90 Acute Care Surgery Unit, Ospedale G. Da Saliceto, Piacenza, Italy; 91 Chirurgia Generale dell’Ospedale Sant’Anna di San Fermo della Battaglia, San Fermo Della Battaglia, Italy; 92 U.O. Chirurgia Generale PO Trapani, Bergamo, Italy; 93 Uoc Chirurgia Ospedale Santa Maria degli Angeli Adria, Adria, Italy; 94 U.O.C. Chirurgia Generale 1 IRCCS Policlinico San Matteo Pavia, University of Pavia, Pavia, Italy; 95 Department of General and Emergency Surgery – “Vito Fazzi” Hospital, Lecce, Italy; 96 General and Emergency Surgery Unit, Emergency Dept, Ospedale Santo Spirito in Sassia, 00193 Rome, Italy; 97 G.Fornaroli” Hospital, Magenta ASST Ovest Milanese, Milan, Italy; 98 Istituto Clinico San Gaudenzio – Novara, Novara, Italy; 99 San Marco Hospital GSD, Zingonia, BG Italy; 100 Unità di Chirurgia GeneraleOspedale Pederzoli, Peschiera del Garda, VR Italy; 101 Emergency and Trauma Surgery, Bufalini Hospital, Cesena, Italy; 102 S.C. Chirurgia Generale ed Epatobiliopancreatica, E.O. Ospedali Galliera, Mura delle Cappuccine 14, 16128 Genoa, Italy; 103 ASST Cremona, Cremona, Italy; 104 Colorectal Surgery Unit, Fondazione Policlinico Campus Bio-Medico, Università Campus Bio-Medico di Roma, Rome, Italy; 105 S.C. Chirurgia Generale Ospedale Di Gardone Val Trompia - ASST Spedali Civili, Brescia, Italy; 106 Unit of Critical Care Surgery and Trauma-Trauma Team University Hospital Careggi, Florence, Italy; 107 ASST Monza, Chirurgia 1, Monza, Italy; 108 Ospedale Santa Maria delle Croci Ravenna Reparto di Chirurgia d’Urgenza, Ravenna, Italy; 109 UOC Chirurgia GeneraleOspedale Fatebenefratelli e Oftalmico, ASST Fatebenefratelli Sacco, Milan, Italy; 110 General Surgery Unit, San Paolo Hospital, Civitavecchia, Italy; 111 Department of Medical and Surgical Sciences, University of Foggia, Foggia, Italy; 112 General Surgery Unit, Infermi Hospital, Rimini, Italy; 113 IRCCS Azienda Ospedaliero Universitaria di Bologna, Bologna, Italy; 114 Alma Mater Studiorum University of Bologna, Bologna, Italy; 115 University of Naples “Federico II”, Naples, Italy; 116 General Surgery,Emergencies and New Technologies, Baggiovara Civil Hospital Modena, Baggiovara, Italy; 117 General Surgery, Ospedale Papa Giovanni XXIII, ASST Papa Giovanni XXIII, Bergamo, Italy; 118 Chirurgia Generale e OncologicaOsp. Mauriziano – Torino, Turin, Italy; 119 General Surgery Unit - ASST Valtellina e Alto Lario, Sondrio Hospital – Sondrio, Sondrio, Italy; 120 Chiba Emergency Medical Center, Chiba, Japan; 121 Tenwek Hospital, Bomet, Kenya; 122 Department of Surgery Hospital of Lithuanian University of Health Sciences, Kaunas, Lithuania; 123 Clinic of Gastroenterology, Nephrourology, and Surgery, Institute of Clinical Medicine, Faculty of Medicine, Vilnius University, Vilnius, Lithuania; 124 Department of Surgery, School of Medical Sciences and Hospital, Universiti Sains Malaysia, Gelugor, Malaysia; 125 Department of General Surgery, Hospital Sultan Ismail Petra, Kuala Krai, Kelantan Malaysia; 126 Departamento de Cirugía Gastrointestinal y Enfermedades Digestivas “DR DANIEL RIOS CRUZ”. Hospital Center Vista Hermosa, Cuernavaca Morelos, México; 127 Hospital Regional de Alta Especialidad del Bajio, León, Mexico; 128 Hospital Santo Tomás, Panamá City, Panamá; 129 Department of General Surgery, Jagiellonian University Medical College, Krakow, Poland; 130 Department of General, Oncological, Metabolic and Thoracic Surgery, Military Institute of Medicine, Warsaw, Poland; 131 Centro Hospitalar de Vila Nova de Gaia e Espinho, EPE, Vila Nova de Gaia, Portugal; 132 Hospital Professor Doutor Fernando Fonseca, Amadora, Portugal; 133 Unidade Local de Saúde do Nordeste, EPE; Serviço de Cirurgia Geral, Bragança, Portugal; 134 CHTMAD – Portugal,, Vila Real, Portugal; 135 General Surgery Grupo Trofa Saúde, Porto, Portugal; 136 General Surgery Department, Centro Hospitalar Barreiro Montijo, E.P.E., Barreiro, Portugal; 137 Centro Hospitalar Universitário do Algarve - Unidade de Faro, Faro, Portugal; 138 Carol Davila University of Medicine and Pharmacy Bucharest and Elias University Emergency Hospital Bucharest, Bucharest, Romania; 139 Department of Abdominal Surgery, Vladimir City Emergency Hospital, Vladimir, Russia; 140 Department of Surgical Diseases No. 3, Gomel State Medical University, University Clinic, Gomel, Belarus; 141 Pirogov Medical University Research Institute of Clinical Surgery, Moscow, Russia; 142 Forth Valley Royal Hospital, Larbert, Scotland, UK; 143 4t, Department of Surgery, University Hospital Bratislava, Bratislava, Slovakia; 144 1s, Department of Surgery, UPJS and UNLP Kosice, Kosice, Slovak Republic; 145 Gastrointestinal Surgery Department, Hospital Clinic de Barcelona, C/Villarroel 170, 08036 Barcelona, Spain; 146 Policlinico Umberto I University Hospital - Sapienza University of Rome, Rome, Italy; 147 Division of Surgical Oncology and Digestive Surgery, Department of Oncology, San Luigi University Hospital, University of Turin, 10043 Orbassano (Turin), Italy; 148 Ospedale Civile Spirito Santo, Pescara, Italy; 149 Emergency Surgery Unit, Hospital Virgen del Rocío, Seville, Spain; 150 General and Colorectal Surgeon, Hospital General Universitario de Ciudad Real, Ciudad Real, Spain; 151 General and Digestive Surgery. Virgen de la Arrixaca University Hospital (IMIB-Arrixaca), Murcia, Spain; 152 Emergency Surgery Unit, University Hospital of Gran Canaria Dr Negrin, Las Palmas de Gran Canaria, Spain; 153 Hospital Universitario Príncipe de Asturias, Alcalá de Henares, Madrid, Spain; 154 Colorectal Surgery, Badajoz University Hospital, Badajoz, Spain; 155 Donostia University Hospital, San Sebastián, Spain; 156 Emergency Surgery Unit, Department of General Surgery, Hospital del Mar, Barcelona, Spain; 157 Department of Surgery, Miguel Servet University Hospital, Saragossa, Spain; 158 Hospital Clínico San Carlos in Madrid, Madrid, Spain; 159 Hospital Universitari Son Llàtzer, Palma, Spain; 160 Hospital General Universitario Gregorio Marañón, Madrid, Spain; 161 Hospital General Universitario de Alicante, Instituto de Investigación Sanitaria y Biomédica de Alicante (ISABIAL), Alicante, Spain; 162 Hospital Arnau de Vilanova, Lleida, Cataluña Spain; 163 Trauma and Emergency Surgery Unit General, Digestive and Transplantation Department, University Regional Hospital Málaga, Málaga, Spain; 164 Colorectal and Gastrointestinal Department Hospital General Universitario de Elche Universidad Miguel Hernández, Elche, Alicante Spain; 165 Department of General and Digestive Surgery. Hospital, Universitario Doctor Peset, Valencia, Spain; 166 Hepatobiliar and Pancreatic Surgery Unit General and Digestive Surgery University Hospital Virgen de Valme, Seville, Spain; 167 Emergency Surgery Unit at Hospital Universitari Parc Tauli, Sabadell, Spain; 168 General and Colorectal Surgeon, Hospital Universitario Infanta Sofia Madrid, Madrid, Spain; 169 General Surgery Service of the University Hospital of Salamanca, Salamanca, Spain; 170 Hospital Universitario Torrecardenas Almeria, Almeria, Spain; 171 Hospital Universtario Puerta de Hierro, Madrid, Spain; 172 Hospital Universitario de Cabueñes Gijón, Asturias, Spain; 173 HPB and Transplantation Unit, Head of Emergency Surgery Unit, Seville, Spain; 174 General and Digestive Surgery Department, Reina Sofía University Hospital, Cordoba, Spain; 175 Hospital Central de la Defensa Gómez Ulla, Madrid, Spain; 176 Colorectal Surgery, Vall d’Hebron University Hospital, Barcelona, Spain; 177 Department of Sugery, Almoalem Medical City, Khartoum, Sudan; 178 Colorectal Surgery Unit, Department of Surgery and Faculty of Medicine Siriraj Hospital, Mahidol University, Bangkok, 10700 Thailand; 179 Department of General Surgery Bizerte Hospital, Faculty of Medicine of Tunis, University Tunis El Manar, Tunis, Tunisia; 180 Department of Gastrointestinal Surgery, Sahloul Hospital, Sousse, Tunis, Tunisia; 181 Sfax Medical School, Tunis, Tunisia; 182 Samsun Training and Research Hospital Colak, Samsun, Turkey; 183 Department of General Surgery, Division of Upper GI Surgery and Institute of Medical Science, Department of Biostatistics and Medical Informatics, Trabzon, Turkey; 184 Department of General Surgery, Sakarya University Training and Research Hospital, Sakarya, Turkey; 185 Department of General Surgery, Sakarya University Faculty of Medicine, Sakarya, Turkey; 186 General Surgery Clinic, School of Medicine, Erzincan Binali Yıldırım University, Erzincan, Turkey; 187 Private Ortadogu Hospital Adana, Adana, Turkey; 188 General Surgery Clinic, Health Sciences University, Kanuni Sultan Süleyman Training and Research Hospital, Istanbul, Turkey; 189 University of Health Sciences, Adana City Training and Research Hospital, Adana, Turkey; 190 Department of General Surgery, University of Health Sciences Tepecik Training and Research Hospital, İzmir, Turkey; 191 Department of General Surgery Elazig, Elazig Health Practice and Research Center, Elazig, Turkey; 192 Department of General Surgery, Bursa Kestel State Hospital, Kestel, Turkey; 193 Department of General Surgery, Bagcilar Training and Research Hospital University of Health Science, Istanbul, Turkey; 194 Department of General Surgery, Koç University Hospital, Istanbul, Turkey; 195 John Radcliffe Hospital Oxford University Hospitals NHS Foundation Trust, Oxford, UK; 196 Brighton and Sussex University Hospitals, Brighton, UK; 197 Northwick Park and St Mark’s Hospital – London North West NHS Trust, Harrow, UK; 198 Hospital de ClínicasClínica Quirúrgica ¨F¨, Montevideo, Uruguay; 199 Department of Surgery, School of Medicine, Westchester Medical Center, New York Medical College, Valhalla, USA; 200 Comparative Effectiveness and Clinical Outcomes Research Center, Riverside University Health System Medical Center, Moreno Valley, CA USA; 201 Massachusetts General Hospital and Harvard Medical School, Boston, MA USA; 202 Division of Trauma, Acute Care Surgery and Surgical Critical Care, Department of Surgery, West Virginia University, Morgantown, USA; 203 Crozer-Chester Medical Center, Upland, PA USA; 204 Cooper University Hospital, Camden, NJ USA; 205 University of Michigan, Ann Arbor, USA; 206 Department of Surgery, University of Missouri, Columbia, USA; 207. Joseph Mercy, Ann Arbor, MI USA; 208 Scripps Memorial Hospital La Jolla, San Diego, USA; 209 General, Emergency and Trauma Surgery Department, Pisa University Hospital, Pisa, Italy; 210 Department of Surgery, Macerata Hospital, Macerata, Italy; 211 Healthcare Administration, Santa Maria Delle Croci Hospital, AUSL Romagna, Ravenna, Italy; 212 General Surgery, Department of Clinical and Experimental Sciences, University of Brescia, Brescia, Italy

**Keywords:** Hartmann’s procedure, Ostomy, Emergency surgery, Resection, Primary anastomosis, Left side, Colon, Diverticulitis, Colorectal cancer

## Abstract

**Background:**

Literature suggests colonic resection and primary anastomosis (RPA) instead of Hartmann’s procedure (HP) for the treatment of left-sided colonic emergencies. We aim to evaluate the surgical options globally used to treat patients with acute left-sided colonic emergencies and the factors that leading to the choice of treatment, comparing HP and RPA.

**Methods:**

This is a prospective, international, multicenter, observational study registered on ClinicalTrials.gov. A total 1215 patients with left-sided colonic emergencies who required surgery were included from 204 centers during the period of March 1, 2020, to May 31, 2020. with a 1-year follow-up.

**Results:**

564 patients (43.1%) were females. The mean age was 65.9 ± 15.6 years. HP was performed in 697 (57.3%) patients and RPA in 384 (31.6%) cases. Complicated acute diverticulitis was the most common cause of left-sided colonic emergencies (40.2%), followed by colorectal malignancy (36.6%). Severe complications (Clavien-Dindo ≥ 3b) were higher in the HP group (*P* < 0.001). 30-day mortality was higher in HP patients (13.7%), especially in case of bowel perforation and diffused peritonitis. 1-year follow-up showed no differences on ostomy reversal rate between HP and RPA. (*P* = 0.127). A backward likelihood logistic regression model showed that RPA was preferred in younger patients, having low ASA score (≤ 3), in case of large bowel obstruction, absence of colonic ischemia, longer time from admission to surgery, operating early at the day working hours, by a surgeon who performed more than 50 colorectal resections.

**Conclusions:**

After 100 years since the first Hartmann’s procedure, HP remains the most common treatment for left-sided colorectal emergencies. Treatment’s choice depends on patient characteristics, the time of surgery and the experience of the surgeon. RPA should be considered as the gold standard for surgery, with HP being an exception.

## Introduction

The Hartmann’s procedure (HP) is a rapid, simple surgical procedure, with relatively low perioperative morbidity and mortality. It was first described in 1921 as a solution for obstructed left-sided colonic carcinomas. Hartmann’s procedure consists of 3 steps: (1) resection of a diseased segment of the colon near the rectosigmoid junction, (2) closure of the distal rectal stump and (3) formation of an end colostomy [1–3].

During early 1900s three staged approach (first stage, diverting colostomy; second stage, resection of the diseased colon; third and last stage, colostomy closure) was the most common treatment for left-sided colonic diseases. Since the second half of the last century thanks to the discovery of antibiotics, the surgical practice changed, enabling surgeons to control postoperative infections and HP started to be used [4].

HP showed better outcomes than three-stage surgery due to less postoperative peritonitis, fewer reoperations, and lower mortality. In the 1980s and 1990s, different studies favored HP, becoming the first-line treatment for left-sided colonic emergencies [5, 6] However, in the last 2 decades, the role of HP has been questioned compared with colonic resection and primary anastomosis. [7, 8] There was no difference in major postoperative complications and mortality between these two procedures [8–10]. Furthermore, the presence of fecal peritonitis was no longer considered an absolute contraindication for immediate bowel continuity reconstruction. [6–10]

Furthermore, only few patients get their stoma reversed after HP. Hartmann’s reversal is also associated with high morbidity rates up to 58% and mortality up to 3.6%, with non-reversal rate ranging from 23 to 74% [11].

Despite the growing evidence supporting primary anastomosis for left-sided colonic emergencies, many surgeons are still reluctant to follow this evidence. The main concern is the anastomotic leakage which can be disastrous, especially in sick patient, leading to medicolegal implications. Other factors may affect the choice of HP over other treatment, most of these procedures are typically performed beyond normal working hours, and often by young surgeons. [12–14]

The Goodbye Hartmann Trial aimed to evaluate the surgical options globally used to treat patients with acute left-sided colonic emergencies and the factors impacting treatment choice, comparing HP and RPA.

## Methods

### Study design

This study was a multicenter, prospective, observational study done in 204 hospitals from 31 different countries in 5 continents.

The study was developed and presented, according to the Strengthening the Reporting of Observational Studies in Epidemiology (STROBE) guidelines. The study was conducted in accordance with the principles of the Declaration of Helsinki and Good Epidemiological Practices [15].

The study was approved by an independent ethical committee (Comitato etico AVEN – area vasta Emilia nord) and by the local ethical committees of all participating centers. Written informed consent was obtained from all patients. The participating surgeons performed their duties according to their usual practices.

The Goodbye Hartmann Trial was registered in ClinicalTrials.gov (ID: NCT04829032).

Data were collected and managed using REDCap electronic data capture tools hosted at Parma University Hospital. [16, 17] The recruitment period lasted 3 months (March 1 2021, to May 31 2021).

No patient’s identifiable data (name, date of birth, address, telephone number, etc.) were recorded.

### Patient selection criteria

Inclusion criteria: patients aged between 18 and 100 years; diagnosis of left-sided (splenic flexure, descending colon and sigmoid colon) colonic emergency (perforated diverticulitis with purulent or fecal peritonitis; large bowel perforation-obstruction; colon cancer perforation-obstruction; ischemic colitis; abdominal trauma); surgical treatment with RPA, HP, ileostomy or colostomy.

Exclusion criteria: patients ineligibile for surgery, hemodynamically unstable patients, defined as patients with an abnormal or unstable blood pressure that resulted in tissue hypoperfusion; patients with left-sided colonic emergency managed with non-surgical treatment; patients with previous colorectal surgery; patients with concomitant non colonic emergencies.

### Variables and definitions

Demographic data and baseline characteristics: age, gender, BMI, comorbidities, ASA score, previous abdominal surgery, Glasgow Coma Scale, quick Sequential Organ Failure Assessment score (qSOFA) [18], symptoms. Vital parameters: temperature, systolic blood pressure, respiratory rate. Laboratory data: white cell blood count (WBC), blood hemoglobin concentration, C-reactive protein (CRP). Disease characteristics: etiology (acute complicated diverticulitis, colorectal cancer, colon ischemia, abdominal trauma, foreign bodies, volvulus, intussusception); preoperative diagnosis and assessment was performed according to the clinical practice of each center, CT scan of the abdomen was always performed in case of diverticulitis and the severity was assessed according to 2015 CT driven classification of left colon acute diverticulitis [19]; clinical presentation (perforation, obstruction, ischemia).

Surgical details: Hartmann’s procedure (HP), colonic resection with primary anastomosis with or without diverting stoma (RPA), stoma without colonic resection.

Hospital characteristics: hospital type, annual volume of emergency surgical procedures; annual volume of surgical left-sided colonic disease; availability of Intensive Care Unit (ICU)). Surgeon’s experience. Time of surgery: weekdays, weekend, bank holidays, night shift. Postoperative outcomes: length of stay (LOS), Clavien-Dindo Classification, reoperation, anastomotic leakage. Follow-up data was collected in all patients at 1 year after the index admission, including data on subsequent stoma reversal and related complications.

### Outcomes

The primary objective was to analyze the factors leading to the surgical choice.

Secondary aims included defining the rate of Hartmann’s procedure reversal and the rate of permanent stoma after 1 year of follow-up.

### Statistical analysis

Patients were divided into three main groups: patients who underwent Hartmann’s procedure; patients who underwent colonic resection with primary anastomosis with or without ileostomy; and patients who underwent only ostomy (ileostomy or colostomy) without colonic resection, according to the most common treatment performed in left-sided colonic emergencies. Subgroup analysis was performed for patients with colorectal cancer and those with complicated acute diverticulitis. Quantitative data was expressed as mean (SD) or median and interquartile range (IQR, minimum and maximum values). The qualitative data were presented as absolute frequencies, relative frequencies, cumulated frequencies, and percentages. Student’s t test, Mann Whitney U test or ANOVA were used for comparisons of continuous or ordinal variables among groups as appropriate. Chi-squared test or Fisher’s exact test, as appropriate, was used for analysis of categorical data.

A logistic regression model defining the factors affecting the decision to do primary anastomosis was performed. The patients were divided into two groups: those who had resection and primary anastomosis of the colon (n = 384) and those who had Hartman’s procedure or ostomy alone (n = 831). Variables who had a loose p value of less than 0.1 were entered into a backward Stepwise (Likelihood Ratio) logistic regression model defining factors affecting the decision to perform resection and primary anastomosis of the colon. Data analysis was performed using IBM SPSS Statistics 26.0. A p value of less than 0.05 was accepted as significant.

## Results

### Baseline patient characteristics

A total 1307 patients were included in the study.

Complete data were available in 1215 (92.9%) patients. HP was performed in 697 (57.3%) patients, RPA in 384 (31.6%) cases and ostomy (ileostomy or colostomy) without bowel resection in 134 (11.0%) patients.

The baseline characteristics of the study cohort stratified according to the surgical procedure are reported on Table 1.

**Table 1:** Baseline characteristics

Variable	Total	HP group	RPA group	Ostomy group	*P* value
Age—mean ± SD	65.8 ± 15.6	68.7 ± 15.0	61.7 ± 14.9	62.5 ± 17.5	< .001
Female sex—N. (%)	557 (45.9%)	331 (47.5%)	168 (43.8%)	58 (43.6%)	0.431
Body Mass Index (BMI)—Mean ± SD	26.5 ± 4.9	26.5 ± 5.2	26.8 ± 4.4	25.7 ± 4.9	0.123
*ASA—N.* (%)					< .001
1	144 (11.9%)	71 (10.2%)	55 (14.3%)	18 (13.6%)	
2	451 (37.3%)	214 (30.8%)	189 (49.4%)	48 (36.3%)	
3	451 (37.3%)	274 (39.5%)	128 (33.5%)	49 (37.1%)	
4	138 (11.4%)	113 (16.3%)	10 (2.6%)	15 (11.3%)	
5	23 (1.9%)	21 (3.0%)	0 (0.0%)	2 (1.5%)	
Previous abdominal surgery—N. (%)	373 (30.9%)	220 (31.6%)	118 (30.7%)	35 (26.3%)	0.480
Fever—N. (%)	285 (23.4%)	193 (28.9%)	71 (19.3%)	21 (16.1%)	< .001
qSOFA score ≥ 2—N. (%)	142 (11.6%)	105 (15.0%)	26 (6.7%)	11 (8.2%)	< .001
WBC (10^9/L)—Mean ± SD	12.8 ± 7.8	13.3 ± 9.2	12.0 ± 4.8	12.6 ± 6.6	0.048
HB—mean ± SD	12.3 ± 3.6	12.2 ± 4.2	12.7 ± 2.7	11.7 ± 2.1	0.021
CRP—mean ± SD	85.1 ± 88.9	93.4 ± 93.1	73.6 ± 81.8	70.7 ± 78.0	0.001

HP, Hartmann’s procedure; RPA, primary anastomosis

### Disease characteristics

Acute complicated diverticulitis (ACD) and colorectal cancer (CRC) were the most common causes of left-sided colonic emergencies. CRC and ACD patients’ characteristics are reported in Appendix 1 and 2.

HP was performed mostly in presence of large bowel perforations (455/694, 65.5%). RPA was performed prevalently in large bowel obstruction (189/384 (49.2%) (*P* < 0.001), Table 2.

**Table 2:** Disease types and clinical presentation

Variable	Total	HP group	RPA group	Ostomy group	*P* value
*Etiology*					
Complicated Acute Diverticulitis	490 (40.3%)	304 (43.6%)	168 (43.7%)	18 (13.4%)	< .001
CRC	445 (36.6%)	229 (32.8%)	154 (40.1%)	62 (46.2%)	0.003
Sigmoid volvulus	55 (4.5%)	31 (4.4%)	16 (4.1%)	8 (5.9%)	0.681
Foreign body	21 (1.7%)	14 (2.0%)	7 (1.8%)	0 (0.0%)	0.833
Trauma	21 (1.7%)	7 (1.0%)	8 (2.0%)	6 (4.4%)	0.015
Intussusception	5 (0.4%)	2 (0.2%)	1 (0.2%)	2 (1.4%)	0.117
Other cancer	15 (1.2%)	5 (0.7%)	2 (0.5%)	8 (5.9%)	< .001
Other	163 (13.3%)	105 (15.0%)	28 (7.2%)	30 (22.3%)	< .001
*Clinical presentation*					
Large bowel perforation	694 (57.1%)	455 (65.5%)	201 (28.9%)	38 (5.4%)	< .001
Large bowel obstruction	527 (43.7%)	255 (37.0%)	189 (49.2%)	83 (62.4%)	< .001
Colonic Ischemia	119 (9.9%)	91 (13.2%)	13 (3.4%)	15 (11.1%)	< .001

In patients with large bowel perforation HP was preferred especially in patients with ASA score ≥ 3 (OR = 1.49; *P* = 0.002), within 12 h from hospital admission (OR = 0.64; *P* = 0.047) and during nighttime (OR = 1.73; *P* = 0.013).

In patients with large bowel obstruction, HP was preferred in patients with ASA score ≥ 3 (OR = 1.32; *P* = 0.028), within 12 h from hospital admission (OR = 0.65; *P* = 0.029), during nighttime (OR = 2.16; *P* = 0.000) and in centers with low volume of emergency surgical procedures (OR = 0.62; *P* = 0.023).

### Time of surgery

HP was generally performed within 12 h from hospital admission in 396 (56.7%) patients Conversely, the 40.1% of RPA cases were performed after 24 h from hospital admission (*P* < 0.001).

Hospital’s characteristics didn’t affect the time from hospital admission to surgery (*P* = 0.285).

During weekends, HP was the most performed procedure (178/270, 65.9%). RPA was performed only in 64/270 patients (23.7%) during weekends (*P* = 0.025).

HP distribution during daytime and nighttime was similar with the 51.5% of HP performed during the day and the 48.5% during the night (from 8 pm to 7am). Conversely, most of RPA (73.4%) were performed during the day and only the 26.6% of RPA during the night (*P* < 0.001).

HP was the most common treatment during weekends and nighttime also in patients with low ASA score. During the weekends and the nighttime, the 59.3% and 62.2% of ASA < 3 patients respectively underwent HP against the 44.7% of weekdays and 39.6% of daytime (*P* = 0.013) (Table 3).

**Table 3:** Time of surgery

Variable	Total	HP group	RPA group	Ostomy group	*P* value
*Time from hospital admission to surgery*					< .001
Less than 1 h	37 (3.0%)	25 (3.5%)	8 (2.0%)	4 (2.9%)	
Between 1 and 6 h	402 (33.1%)	255 (36.6%)	99 (25.8%)	48 (35.8%)	
From 6 to 12 h	213 (17.5%)	141 (20.2%)	54 (14.0%)	18 (13.4%)	
From 12 to 24 h	169 (13.9%)	90 (12.9%)	68 (17.7%)	11 (8.2%)	
After 24 h	392 (32.3%)	185 (26.5%)	154 (40.2%)	53 (39.5%)	
*Day of surgery*					0.025
Weekday	926 (76.2%)	507 (72.7%)	314 (81.7%)	105 (78.3%)	
Weekend	270 (22.2%)	178 (25.5%)	64 (16.6%)	28 (20.8%)	
Public holiday	18 (1.4%)	12 (1.7%)	5 (1.3%)	1 (0.7%)	
*Time of surgery*					< .001
Day: 7 am–8 pm	735 (60.4%)	359 (51.5%)	282 (73.4%)	94 (70.1%)	
Early night: 8 pm–11 pm	241 (19.8%)	157 (22.5%)	62 (16.1%)	22 (16.4%)	
Late night: 11 pm–7am	238 (19.6%)	181 (25.9%)	39 (10.1%)	18 (13.4%)	

### Surgical approach

Laparotomy was the most common surgical approach (n = 985, 81.1%). Among the different surgical procedures, 623 (89.5%) HP and 110 (82.0%) ostomies were performed via laparotomy. Laparoscopic colonic resection with primary anastomosis was the most common laparoscopic procedure (127/233, 54.5%) (*P* < 0.001). Peculiarly, 80.7% of laparoscopic procedures were performed during daytime (*P* < 0.001). During nighttime, laparoscopy was performed only in 43/478 (8.9%) patients, 28 (12.5%) during early night and only in 15 (6.7%) in late night. Robotic surgery was attempted 6 times (0.49%), only one patient underwent robotic HP while the other five underwent robotic RPA.

### Surgeon and center characteristics

Inexperienced surgeons performed more HP and ostomies than RPA compared with experienced surgeons (*P* < 0.001). Inexperienced surgeons performed less laparoscopic procedures (12.6%) than experienced surgeons (20.6%) (*P* = 0.041). Inexperienced surgeons also performed 50.1% of operations during nighttime (11 pm to 7 pm). Notably the 29.9% of all surgical procedures took place in the late night (from 11 pm to 7 am). While experienced surgeons performed 35.4% of operations during nighttime and only 16.1% during late night (*P* = 0.002).

The distribution and types of surgical procedures were similar across the hospitals, regardless of origin country. Most of the RPA cases were performed in hospitals with high volume of emergency surgical procedures. (*P* = 0.005). The surgeon and center characteristics are summarized on Table 4.

**Table 4:** Surgeon and center characteristics

Variable	Total	HP group	RPA group	Ostomy group	*P* value
*Surgeon’s experience*					< .001
> 50 colorectal resections	901 (74.1%)	497 (55.1%)	322 (35.7%)	82 (9.1%)	
< 50 colorectal resections	314 (25.8%)	200 (63.6%)	62 (19.7%)	52 (16.5%)	
> 10 colorectal resections per year in the last 5 years	871 (71.6%)	491 (56.3%)	303 (34.7%)	77 (8.8%)	< .001
< 10 colorectal resections per year in the last 5 years	330 (27.1%)	199 (60.3%)	77 (23.3%)	54 (16.3%)	
*Center characteristics*					0.004
Academic	568 (47.2%)	313 (45.4%)	180 (47.2%)	75 (56.3%)	
Trauma Center	112 (11.8%)	73 (10.6%)	33 (8.6%)	6 (4.5%)	
Non-Trauma Center	535 (41.0%)	311 (44.0%)	171 (44.2%)	53 (39.2%)	
Presence of Intensive Care Unit (ICU)?	1198 (98.5%)	683 (99.1%)	374 (97.6%)	131 (98.4%)	0.170
*Annual volume of emergency surgical procedures*					0.005
< 500	265 (22.0%)	155 (58.4%)	82 (30.9%)	28 (10.5%)	
Between 500 and 1000	466 (38.7%)	290 (62.2%)	133 (28.5%)	43 (9.2%)	
> 1000	473 (39.2%)	244 (51.5%)	167 (35.3%)	62 (13.1%)	
*Annual volume of elective colorectal resections*					0.245
< 50	72 (5.9%)	41 (56.9%)	21 (29.1%)	10 (13.8%)	
Between 50 and 100	386 (32.0%)	237 (61.3%)	109 (28.2%)	40 (10.3%)	
> 100	747 (61.9%)	412 (55.1%)	252 (33.7%)	83 (11.1%)	

### Postoperative outcomes

Length of stay (LOS) was higher in HP group (13.4 ± 12.1 days) compared with the RPA group (11.7 ± 10.2 days) (*P* = 0.048).

LOS was higher in patients treated with laparotomy (13.4 ± 11.8 days) compared to patients treated with laparoscopic approach (9.8 ± 7.0 days). (*P* < 0.001).

Postoperative complications were higher in patients who underwent HP (*P* < 0.001).

Severe complications (Clavien-Dindo ≥ 3b) were higher in HP group (*P* < 0.001).

Severe complications in ASA score < 3 were lower in RPA group than HP group, also in case of perforation and diffuse peritonitis (*P* = 0.017).

Severe complications in patients with ASA score > 3 were similar in both HP and RPA groups. (*P* > 0.05).

Severe complications were higher in high-risk patients (diffuse peritonitis, qSOFA score ≥ 2) with ASA score ≥ 3 (*P* = 0.002).

Mortality was significantly higher in patients with bowel perforation and diffused peritonitis (*P* < 0.001).

Anastomotic leakage was reported in 46 patients (11.9%). Conservative treatment of anastomotic leakage was effective in 10 patients (21.6%), in the other 36 cases, surgery was required to manage anastomotic leak (78.4%).

Postoperative outcomes are summarized in Table 5.

**Table 5:** Postoperative outcomes

Variable	Total	HP group	RPA group	Ostomy group	*P* value
LOS—mean ± SD	12.7 ± 11.1	13.4 ± 12.1	11.7 ± 10.2	12.0 ± 8.4	0.048
Complications	554 (46.7%)	352 (51.9%)	146 (38.8%)	56 (42.1%)	< .001
*Clavien-Dindo classification*					0.002
1	237 (32.2%)	129 (19.3%)	86 (23.8%)	22 (17.0%)	
2	246 (20.5%)	151 (22.6%)	72 (20.0%)	23 (17.8%)	
3a	46 (3.9%)	29 (4.3%)	13 (3.6%)	4 (3.1%)	
3b	89 (7.6%)	46 (6.8%)	32 (8.8%)	11 (8.5%)	
4a	31 (2.6%)	21 (3.1%)	7 (1.9%)	3 (2.3%)	
4b	19 (1.6%)	10 (1.4%)	7 (1.9%)	2 (1.5%)	
30-day mortality	115 (9.9%)	92 (13.7%)	9 (2.5%)	14 (10.8%)	< .001

### Follow-up

Only 21.6% of HP patients underwent surgery for ostomy reversal during the 1-year follow-up, against the 64.7% of RPA patients. Complication rate after ostomy reversal was higher in the HP groups (*P* = 0.41). Anastomotic leakage was 7.5% in HP group compared with the 9.0% in RPA group (*P* > 0.05). Permanent stoma was reported in 430 (78.3%) cases in HP group, similar to the ostomy group with 77 cases (76.2%). In the RPA group only 22 (6.6%) patients had a stoma after 1-year from surgery (*P* < 0.001) (Table 6).

**Table 6:** 1-year follow-up of patients with ostomy

Variable	Total	HP group	RPA group	Ostomy group	*P* value
Permanent stoma	760 (82.9%)	430 (78.3%)	22 (6.6%)	77 (76.2%)	< .001
Surgery for ostomy reversal	198 (21.6%)	119 (21.6%)	55 (64.7%)	24 (23.7%)	< .001
Complication during reversal surgery	30 (15.1%)	19 (15.9%)	6 (10.9%)	5 (20.8%)	0.041

### Primary aim: which factors influence the choice of HP and RPA?

The logistic regression model was made dividing patients into 2 groups: primary anastomosis of the colon (n = 384) and Hartman’s procedure or ostomy alone (n = 831).

The logistic regression model was highly significant (*P* < 0.001) having a Nagelkerke R Square of 0.2.

The analysis predicted several factors that contributed to performing RPA instead of HP. (Table 7).

**Table 7:** The backward stepwise (likelihood ratio) logistic regression model defining factors affecting the decision to perform resection and primary anastomosis of the colon, 384 resection and primary anastomosis patients compared with 831 Hartman’s procedure or ostomy patients

Variable	Estimate	S.E	Wald	*P* value	OR	OR 95% C.I lower limit	OR 95% C.I. upper limit
Age	− 0.02	0.006	10.484	0.001	0.98	0.968	0.992
ASA Classification	− 0.45	0.108	17.026	< .001	0.641	0.519	0.792
Large bowel obstruction	0.41	0.169	5.792	0.016	1.501	1.078	2.091
Colonic ischemia	− 1.02	0.361	7.911	0.005	0.362	0.178	0.735
Time from admission to surgery	0.23	0.066	12.210	< 0.001	1.260	1.107	1.434
Earlier Time of day	− 0.26	0.115	5.083	0.024	0.771	.615	0.967
Surgeon Experience (> 50 colorectal resections)	0.67	0.202	10.865	< .001	1.948	1.310	2.897
Constant	0.66	0.510	1.651	0.199	1.926		

OR, odds ratio; SE, standard error; CI, confidence interval

The choice of surgical procedure is related to patient’s factors, etiology, hospital setting and surgeon’s characteristics.

RPA was preferred in younger patients, having low ASA score (≤ 3), in case of large bowel obstruction, absence of colonic ischemia, longer time between admission and surgery, operating early at the day working hours, and by a surgeon who performed more than 50 colorectal resections.

In contrast, HP was the preferred procedure in patients with ASA score status ≥ 3, qSOFA score ≥ 2, in case of large bowel perforation, in low volume hospitals, within 12 h from hospital admission, performed by inexperienced surgeons and during the night.

## Discussion

The results of this study showed that HP remains the most common surgical procedure for colorectal emergencies. Several factors may be related to HP choice. Regression model analysis showed that HP was preferred in low volume hospitals, by inexperienced surgeons, during the night, in older patients, large bowel perforation, colonic ischemia, and patients having ASA score ≥ 3 and qSOFA score ≥ 2.

Typically, the greatest concern against RPA in the treatment of colorectal emergency was anastomotic leakage which ranged from 3.5 to 30% in emergency surgery. [9, 10, 20] In this study anastomotic leakage after RPA was 11.9% out of whom 78.4% required surgery.

In the last decades several studies evidenced no difference in major postoperative complications and mortality between HP and RPA, [7, 9, 12, 14, 21] as also reported in the present study. Furthermore, recent literature showed better postoperative outcome and reduced mortality after RPA even in large bowel perforations with generalized purulent or fecal peritonitis. [9, 10, 22]

The results of the study confirm literature findings, severe complications were 40% higher in HP group than RPA group (25% compared with 10%), the 30-day mortality was 5 times more in HP (13.7% compared with 2.5% of RPA).

Another factor in favor of RPA, as reported in several studies, was the better stoma-free survival compared with the HP patients [9, 10, 20, 23]. In the present study ostomy reversal after 1-year follow-up was only 25% in HP patients compared to the 64.7% in RPA group.

Furthermore, complications after ostomy reversal were 30% higher in the HP group compared to the RPA group (15.9% vs 10.9%) in the present study. Literature findings showed higher morbidity and anastomotic leak rate of Hartmann’s reversal surgery which ranges from 20 to 50% compared with 2 to 7% in RPA [22, 24–27].

Despite these factors in favor of RPA, usually HP patients have more comorbidities and worse clinical presentation compared to RPA patients [28–31]. In this study Hartmann’s procedure was performed mainly in cases of large bowel perforation, ASA score ≥ 3, and qSOFA score ≥ 2.

Hospitals with high volume of emergency surgery (more than 1000 procedures per year) performed less HP procedures (51.5%) compared to small (58.4%) and medium (62.2%) volume hospitals. The lack of some services (24-h specialist coverage and an on-site CT scanner) could contribute to these differences. [32–34]

Surgical experience, early decision, and faster time to emergency surgery affected the intraoperative surgical errors and clinical outcome. During the night, indication for surgery was usually made by those who do not make the surgery [35–37]. In the present study, most surgical operations (73.6%) were performed by experienced surgeons who have done more than 50 colorectal resections. The 63.4% of surgical procedures performed by inexperienced surgeons, were mainly HP, with only 19.8% being RPA. Conversely, experienced surgeons performed HP in 55.1% of the cases and RPA in 35.8%. Inexperienced surgeons performed less laparoscopic procedures (12.6%) compared with experienced surgeons (20.6%) without a difference in morbidity and mortality. These findings were driven by several factors.

65.7% of patients with ASA score status ≥ 3 and 47.8% of ASA score < 3 patients were treated with HP. ASA score status > 3 has been reported as independent risk factor for postoperative complications, especially in high-risk patients with bowel perforation and diffuse peritonitis [38–40].

Similar severe complications after HP and RPA in ASA score status > 3 were reported in this study. RPA patients with ASA score ≤ 3 showed better postoperative outcomes than HP patients.

RPA was suggested in patients with ASA score = 3 and HP in high-risk patients (diffuse peritonitis, qSOFA score ≥ 2) with ASA score = 3.

HP was performed in ASA score ≤ 3 especially during weekends and nighttime. Several HP performed during weekends (59.3%) and nighttime (62.2%) could be avoided in favor of RPA due to the better postoperative outcomes.

16.8% of patients treated during late night had qSOFA score ≥ 2, whilst only 9.1% of patients treated during daytime had qSOFA score > 2. High qSOFA score was associated with organ dysfunction and a mortality of more than10% which favored the HP procedure. [41, 42]

Laparoscopy was performed in 25.2% of the procedures during daytime, and only in 6.7% during the night. LOS was lower in patients treated with laparoscopy which favor minimally invasive surgery even in emergency surgery [43, 44]. Robotic surgery, although performed in few patients, reflects the increased interest in this approach in emergency surgery [45, 46] which should be properly assessed in future studies despite its limitations.

Performing randomized clinical trials comparing HP and RPA can be challenging. The results of this study supported the use of RPA although HP as a treatment of left-sided colonic emergencies is still a viable option. Nevertheless, we must acknowledge that results carried the risk of selection bias depending on the clinical status of the patient, the experience of the surgeon, the setting of the hospitals, including available technologies (robot, SEMS [47], 24-h specialist coverage and an on-site CT scanner) and time in which surgery was done.

## Conclusions

HP remains the most common treatment for left-sided colorectal emergencies. Selection of the type of surgery depends on the time of surgery, the experience of the surgeon, and patient characteristics. The study supports the use of RPA which should be considered as the gold standard for surgery, with HP being an exception. Several factors contributed to the choice of HP over RPA but they are not often related to higher postoperative outcomes.

The RPA was preferred in younger patients age, having low ASA score (≤ 3), in case of large bowel obstruction, absence of colonic ischemia, longer time between admission and surgery, operating early at the day working hours, by a surgeon who performed more than 50 colorectal resections.

## Data Availability

The original dataset generated during the current study is available from the corresponding author on reasonable request.

## Appendix 1

Colo-rectal cancer emergencies characteristics (N = 445).

**Table:**

Variable	HP group (n = 229)	RPA group (n = 154)	Ostomy group (n = 62)	*P* value
Age—mean ± SD	68.0 ± 14.2	65.6 ± 13.9	64.5 ± 15.5	0.119
Female sex—N. (%)	95 (41.6%)	62 (40.5%)	24 (39.3%)	0.940
*ASA—N*. (%)				0.004
1	25 (10.9%)	18 (11.8%)	8 (13.1%)	
2	94 (41.2%)	80 (52.6%)	24 (39.3%)	
3	76 (33.3%)	52 (34.2%)	25 (40.9%)	
4	27 (11.8%)	2 (1.3%)	4 (6.5%)	
5	6 (2.6%)	0 (0.0%)	0 (0.0%)	
qSOFA score ≥ 2- N. (%)	26 (11.3%)	10 (6.4%)	1 (1.6%)	0.029
*Clinical presentation*				
Large bowel perforation	88 (38.4%)	26 (16.8%)	8 (12.9%)	< 0.001
Large bowel obstruction	141 (61.5%)	128 (83.1%)	54 (87.0%)	< 0.001
Colonic Ischemia	12 (5.3%)	9 (5.9%)	1 (1.6%)	0.404
*Abdominal approach*				
Laparoscopy	14 (6.1%)	33 (21.4%)	8 (12.9%)	< 0.001
Laparotomy	215 (93.8%)	119 (77.2%)	54 (87.0%)	
*Time from hospital admission to surgery* 0.006				
Within 12 h	140 (61.1%)	65 (42.2%)	28 (45.1%)	
After 12 h	89 (38.8%)	89 (57.7%)	34 (61.2%)	
*Time of surgery*				
Weekend	52 (22.7%)	31 (20.1%)	13 (20.9%)	0.804
Early night: 8 pm–11 pm	47 (20.5%)	25 (16.2%)	7 (11.2%)	< 0.001
Late night: 11 pm–7 am	49 (21.3%)	13 (8.4%)	8 (12.9%)	
*Surgeon’s experience*				
> 50 colorectal resections	176 (76.8%)	139 (90.2%)	39 (62.9%)	< 0.001
*Center annual volume of emergency surgical procedures*				
> 1000	83 (36.4%)	85 (55.1%)	26 (42.6%)	0.005
*Center annual volume of elective colorectal resections*				
> 100	149 (65.0%)	110 (71.4%)	44 (70.9%)	0.278
*Postoperative outcomes*				
LOS—mean ± SD	11.2 ± 6.9	10.3 ± 6.6	11.8 ± 8.6	0.289
Complications	109 (48.4%)	51 (33.5%)	22 (36.0%)	0.010
Clavien-Dindo ≥ 3b	47 (20.5%)	22 (14.2%)	10 (16.1%)	0.018
30-day mortality	25 (11.0%)	3 (2.0%)	4 (6.6%)	0.002
*1-year follow-up*				
Permanent stoma	131 (40.4%)	12 (36.3%)	37 (67.2%)	< 0.001
Surgery for ostomy reversal	33 (33.6%)	20 (60.6%)	13 (20.0%)	< 0.001
Complication during reversal surgery	5 (15.1%)	3 (15.0%)	2 (15.3%)	0.791

## Appendix 2

Complicated acute diverticulitis characteristics (N = 490).

**Table:**

Variable	HP group (n = 304)	RPA group (n = 168)	*P* value
Age—mean ± SD	69.1 ± 14.7	60.0 ± 13.2	< 0.001
Female sex—N. (%)	156 (51.3%)	77 (45.8%)	0.255
*ASA—N*. (%)			< 0.001
1	29 (9.5%)	25(14.1%)	
2	77 (25.4%)	78 (46.4%)	
3	140 (46.2%)	61 (36.3%)	
4	50 (16.5%)	4 (2.3%)	
5	7 (2.3%)	0 (0.0%)	
qSOFA score ≥ 2- N. (%)	39 (12.8%)	8 (4.7%)	0.005
*Clinical presentation*			
Large bowel perforation	280 (92.7%)	146 (88.4%)	0.123
Large bowel obstruction	29 (9.6%)	24 (14.3%)	0.125
Colonic Ischemia	14 (4.6%)	1 (0.5%)	0.017
*Abdominal approach*			
Laparoscopy	42 (13.8%)	78 (46.4%)	< 0.001
Laparotomy	260 (85.8%)	87 (51.7%)	
*Time from hospital admission to surgery*			0.013
Within 12 h	187 (61.5%)	67 (39.8%)	
After 12 h	117 (38.4%)	101 (60.1%)	
*Time of surgery*			
Weekend	83 (27.3%)	20 (11.9%)	< 0.001
Early night: 8 pm–11 pm	70 (23.0%)	28 (16.7%)	< 0.001
Late night: 11 pm–7am	81 (26.6%)	17 (10.1%)	
*Surgeon’s experience*			
> 50 colorectal resections	211 (69.4%)	134 (79.7%)	0.015
*Center annual volume of emergency surgical procedures*			
> 1000	86 (28.7%)	51 (30.5%)	0.864
*Center annual volume of elective colorectal resections*			
> 100	154 (51.5%)	101 (60.4%)	0.275
*Postoperative outcomes*			
LOS—mean ± SD	14.0 ± 11.6	12.3 ± 9.2	0.108
Complications	155 (52.3%)	66 (40.0%)	0.011
Clavien-Dindo ≥ 3b	66 (21.7%)	25 (14.8%)	0.007
30-day mortality	34 (11.9%)	3 (1.9%)	< 0.001
*1-year follow-up*			
Permanent stoma	131 (51.9%)	6 (14.2%)	< 0.001
Surgery for ostomy reversal	69 (29.3%)	30 (71.4%)	< 0.001
Complication during reversal surgery	12 (17.3%)	2 (6.6%)	0.027
